# Editorial: Dietary Polyphenols for Improving Gut Health: Volume 1

**DOI:** 10.3389/fnut.2021.760917

**Published:** 2021-10-04

**Authors:** Kai Wang, Guiju Sun, Michael A. Conlon, Wenkai Ren, Guan Yang

**Affiliations:** ^1^Institute of Apicultural Research, Chinese Academy of Agricultural Sciences, Beijing, China; ^2^Department of Nutrition and Food Hygiene, School of Public Health, Southeast University, Nanjing, China; ^3^Commonwealth Scientific and Industrial Research Organisation (CSIRO) Health and Biosecurity, Adelaide, SA, Australia; ^4^College of Animal Science, South China Agricultural University, Guangzhou, China; ^5^Department of Infectious Diseases and Public Health, City University of Hong Kong, Kowloon, Hong Kong, SAR China

**Keywords:** polyphenols, immunology, gut microbiome, immune function, gut barrier function, gut motility

Polyphenols are a large subgroup of phytochemicals which are often in abundance in commonly consumed foods such as fruits, vegetables, and grains. Dietary polyphenols are known to have many biological activities, some of which can potentially protect against various chronic diseases and benefit tissues such as the gut. Within the gastrointestinal tract, dietary polyphenols have been shown to benefit colorectal tissue integrity and function, gut bacterial growth and activities, and the immune system. Studies have also shown that gut microbes can often convert polyphenols into secondary bioactive metabolites that can influence health. Our understanding of the role that dietary polyphenols play in modulating gut health continues to grow, and with it the opportunity to translate this into the development of new or improved dietary supplements or therapies. Thus, current scientific research on polyphenols has aroused great interest and significantly attracted the attention of researchers. This Research Topic aimed to expand our knowledge of how dietary polyphenols can maintain or improve intestinal health, with an emphasis on how this could be translated into their use as dietary supplements to prevent disease or therapies to treat disease.

This Research Topic comprises 8 reviews of the current literature, 1 mini-review, and 13 original research articles by experts in the field. These articles selected by the editors will provide our readers with a deeper understanding of the benefits of dietary polyphenols in improving gut health. These papers are summarized as follows.

## Exploring the Gastrointestinal Protective Effects of Polyphenols Based on Foodomics Approaches

Foodomics is emerging as an important high-throughput analysis approach widely applied in food and nutrition studies. Foodomics integrates several analytical platforms and data processing methods for genomic, transcriptomics, proteomics, and metabolomics studies, enabling exploration of the interactions between polyphenols and the gastrointestinal tract at the molecular level. Zhang et al. completed and published a comprehensive state-of-the-art review on the subject, and contributes to our understanding of the gastrointestinal protective effects of polyphenols.

## Polyphenols, Immunity, and Gastrointestinal Inflammatory Disorder

Immune function is closely related to gut health. Dietary polyphenols were found to promote host immunity through different pathways. Gallic acid (GA) is a typical polyphenol compound common in foods such as tea. Yang et al. reviewed how GA influences the gut microbiota and highlights its modulations of the immune response to maintain intestinal health. Another mini-review contributed by Singh et al. described the effects of polyphenol-rich green tea and its main polyphenolic compounds (–)-epigallocatechin-3-gallate (EGCG), in protecting against malignancy and inflammation. They focused on the cellular mechanisms involved in downregulating the calcium signaling of T cell function by EGCG at the post-transcriptional level.

Ma et al. conducted a research study on a similar topic in which they explored the protective effects of EGCG against acute lipopolysaccharide-induced acute injury. They noticed that EGCG gavage reshaped the intestinal microbial community, reversed the changes in the abundance ratio of *Firmicutes*/*Bacteroidetes*, and significantly reduced the abundance of *Enterobacteriales*. Such protective effects were also attributed to EGCG's modulating effects on the metabolism of sphingolipids.

Colitis is a common gastrointestinal inflammatory disorder and is often studied using animal models. The dextran sulfate sodium (DSS)-induced colitis murine model is widely used. Chen et al. and Hou et al. investigated the protective and therapeutic effects of dietary polyphenols on dextran sodium sulfate (DSS)-induced colitis in mice. Chen et al. showed that juglone, a natural polyphenol found in walnut trees, alleviated DSS-induced mice colitis by decreasing intestinal inflammation and reducing oxidative stress. Hou et al. noticed taxifolin, a natural polyphenol be extracted from onions and other vegetables, prevented DSS-induced mice colitis by blunting the NF-κB signaling, enhancing intestinal barrier, and modulating gut microbiota. Another interesting study performed by Yue et al. applied Irinotecan (CPT-11) as the colitis-inducing reagent. The gastrointestinal toxicity caused by CPT-11 strongly limits its anticancer efficacy. They showed the natural flavonoids from *Glycyrrhiza uralensis* fisch corrected general intestinal microbial dysbiosis and fecal metabolic disorders in CPT-11-induced colitis mice and proposed *G. uralensis* diet as an adjuvant for chemotherapy. Yan et al. contributed an original research article which reported the protective effects of chlorogenic acid (a key coffee polyphenol) against indomethacin-induced gastrointestinal inflammation and mucosa damage. Based on cecal microbiota transplantation approaches, they revealed that chlorogenic acid helped maintain intestinal integrity and alleviate inflammatory responses, primarily by inhibiting the growth of Bacteroides and the accumulation of *Bacteroides*-derived LPS.

## Polyphenols and Colorectal Cancer

Colorectal cancer (CRC) is one of the most prevalent cancers in the world. In this topic, Ding et al. and Long et al. reviewed the potential of polyphenols to protect against CRC. They provided comprehensive information on understanding the regulatory mechanisms involved, including modulations of the intestinal microbiota, inflammation, as well as cancer cell proliferation and apoptosis-related signaling. Also, Chen et al. explored the effects of berberine (BBR) on inhibiting colorectal cancer. They showed that BBR induced alterations in the microbiota and their metabolism in AOM/DSS mice. These articles are expected to help in the development of future applications of dietary polyphenols in clinical intervention strategies, especially for CRC therapies and prevention.

## Polyphenols, Gut Microbiota Dysbiosis, and Metabolic Disease

Gut microbiota dysbiosis is closely related to the development of metabolic disease, such as obesity. Zhao et al. found that monosodium glutamate (MSG)-induced abdominal obesity, accompanied by gut microbiota dysbiosis, contributes to neuronal damage in the hypothalamus. Dietary quercetin modulated the gut microbiota, alleviated hypothalamic damage and down-regulated liver RetSat expression, thus ameliorating abdominal obesity. Ding et al. showed that honokiol supplementation improved obesity by regulating the intestinal microbiota and its production of metabolites, and found that sex may be an important factor in determining honokiol activity in obese mice induced by a high-fat diet-induced obese mice.

## Polyphenols and Gastrointestinal Tract

The gastrointestinal tract is the organ within the body, and its actions play a significant role in shaping the functionality, integrity and composition of the gut microbiota. In the gastrointestinal tract, dietary polyphenols remain largely unabsorbed and accumulate in the large intestine, where they can undergo metabolism by intestinal microbes. In their article, Ray and Mukherjee describe the fates of dietary polyphenols within the gastrointestinal tract and link this with intestinal microbial ecology, biological activities, and human well-being and disease.

## Polyphenols and the Gut-Kidney Axis

Recent studies suggest that there is a potential link between the development of chronic kidney disease (CKD) and functional disorders of the gastrointestinal system. A review presented by Bao et al. showed that polyphenols, such as anthocyanin, catechin, chlorogenic acid, and resveratrol, can regulate the intestinal microbiota. These polyphenols can reduce kidney injury by inhibiting pathogenic bacteria and decreasing host inflammation.

## Polyphenols and the Gut-Liver Axis

Liver diseases represent a major health burden worldwide. The gut microbiome is often involved in liver diseases and may act via the so-called gut-liver axis. Fu et al. showed that a high-fat diet promotes macrophage-mediated hepatic inflammation and aggravates diethylnitrosamine-induced hepatocarcinogenesis in mice. A study by He et al. showed Holly (*Ilex latifolia* Thunb.) polyphenol extracts could alleviate hepatic damage caused by diquat, a powerful herbicide with strong hepatotoxicity, in challenged piglets. Another study by Luo et al., showed polyphenol components of *triadica cochinchinensis* honey (TCH) improved alcohol-induced liver disease by reshaping the gut microbiota of mice.

## Polyphenols as Antibiotic Substitute in Farm Animals

Farmed animals remain the main human-consumed sources of protein, and their production requires significant antimicrobial use (AMU). There is an urgent need to find efficient/cost-effective approaches for decreasing AMU, in particular the use of antimicrobials such as antibiotics, which lead to antimicrobial resistance, during farm animal production. Hasted et al. reviewed the immunostimulatory potential of fruits and their extracts in poultry, and showed they had great promise as a replacement for antibiotic applications in poultry production to maintain poultry health and reduce public health risks. A research article contributed by Cao et al. integrated microbiome and metabolomics approaches to revealed that a polyphenol-rich grape seed extract could be used as an antibiotic substitute in broiler chickens. Another research article presented by Feng et al. explored the effects of polyphenol-rich *Ulva prolifera* extract on intestinal oxidative stress in weaned piglets and intestinal porcine epithelial cells (IPEC-J2) challenged with hydrogen peroxide. They showed that *U. prolifera* extract alleviates oxidative stress via Nrf2 signaling, but independent of the AMPK pathway in weaned piglets.

Proanthocyanidins are naturally occurring polyphenols with variable molecular structures and bioactivities. Andersen-Civil et al. reviewed the modulating effects of dietary proanthocyanidins on enteric infections and immunity.

Collectively, the articles in this Research Topic provide novel insight and a comprehensive set of information relating the effects of dietary polyphenols on the activities and health of the gut. These papers are expected to contribute to the development of strategies and products that utilize polyphenols, in the prevention/therapy of the gastrointestinal disorders. The editors hope that this topic will shed a light on future research in this growing and exciting area of nutrition and Microbes/Nutritional Immunology.

## Author Contributions

KW was a guest associate editor of the Research Topic and wrote the paper draft. GS, MC, GY, and WR served as guest associate editors of this Research Topic and edited the text. All authors listed have made a substantial, direct and intellectual contribution to the work, and approved it for publication.

## Funding

KW was supported by the National Natural Science Foundation of China under Grant 32172791.

## Conflict of Interest

The authors declare that the research was conducted in the absence of any commercial or financial relationships that could be construed as a potential conflict of interest.

## Publisher's Note

All claims expressed in this article are solely those of the authors and do not necessarily represent those of their affiliated organizations, or those of the publisher, the editors and the reviewers. Any product that may be evaluated in this article, or claim that may be made by its manufacturer, is not guaranteed or endorsed by the publisher.

